# The cyclin dependent kinase inhibitor p21^Cip1/Waf1^ is a therapeutic target in high-risk neuroblastoma

**DOI:** 10.3389/fonc.2022.906194

**Published:** 2022-09-06

**Authors:** Agnes Luise Sorteberg, Vesa Halipi, Malin Wickström, Shahrzad Shirazi Fard

**Affiliations:** Department of Women’s and Children’s Health, Karolinska Institutet, Stockholm, Sweden

**Keywords:** p21(Thr145), UC2288, small molecular inhibitor, chemo-resistance, combination treatment, oncogene

## Abstract

Platinum-based chemotherapies such as cisplatin are used as first-line treatment for the paediatric tumour neuroblastoma. Although the majority of neuroblastoma tumours respond to therapy, there is a high fraction of high-risk neuroblastoma patients that eventually relapse with increased resistance. Here, we show that one key determinant of cisplatin sensitivity is phosphorylation of the cyclin-dependent kinase inhibitor p21^Cip1/Waf1^. A panel of eight neuroblastoma cell lines and a *TH-MYCN* mouse model were investigated for the expression of p21^Cip1/Waf1^ using RT-qPCR, Western blot, and immunofluorescence. This was followed by investigation of sensitivity towards cisplatin and the p21^Cip1/Waf1^ inhibitor UC2288. Whereas the cell lines and the mouse model showed low levels of un-phosphorylated p21^Cip1/Waf1^, the phosphorylated p21^Cip1/Waf1^ (Thr145) was highly expressed, which in the cell lines correlated to cisplatin resistance. Furthermore, the neuroblastoma cell lines showed high sensitivity to UC2288, and combination treatment with cisplatin resulted in considerably decreased cell viability and delay in regrowth in the two most resistant cell lines, SK-N-DZ and BE(2)-C. Thus, targeting p21^Cip1/Waf1^ can offer new treatment strategies and subsequently lead to the design of more efficient combination treatments for high-risk neuroblastoma.

## Introduction

Neuroblastoma (NB) is the most common extracranial solid tumour in early childhood. It is a heterogenous neoplasia with clinical presentation ranging from spontaneous regression of metastatic disease to a rapidly progressive course (1, 2). High-risk NB patients, accounting for 50% of all children diagnosed with NB, often present with unresectable primary lesions and/or multiple metastases (1). More importantly, despite intensive multimodal treatment, relapse rates are as high as 50% and are frequently characterised by therapy resistance and intra-tumour diversity, making high-risk NB difficult to cure (3, 4).

In order to combat development of resistant cells, novel targeted strategies are being implemented for high-risk NB, including small-molecule inhibitors of anaplastic lymphoma kinase (ALK), radiolabelled somatostatin analogues, and monoclonal antibodies against the antigen GD2 (5, 6). Even though the overall survival of high-risk NB patients has increased following anti-GD2 immunotherapy, one third of the patients experience treatment failure within the first two years of the treatment, mostly due to a variety of immune evasion strategies (7).

Despite challenges, chemotherapy (CT) continues to be the cornerstone of systemic NB treatment. The platinum complex cisplatin is commonly included in first line therapies as adjuvant therapy, with the aim of inducing cytotoxicity and apoptosis (1, 8, 9). Unfortunately, the effect is not exclusive to cancer cells, and cisplatin-induced side effects include hearing loss, neuro- and/or renal-toxicity or bone marrow-suppression (1, 9, 10). A major role in the response to cisplatin has been ascribed to the tumour cell´s sensitivity to the drug. Recently, the expression of the cyclin-dependent kinase inhibitor p21^Cip1/Waf1^ (p21) has been linked to cisplatin resistance in testicular cancer (11) and in ovarian cancer (12).

The p21 protein is transcriptionally regulated by p53-dependent and –independent pathways and is a key regulator of cell fate. Based on the intracellular localization, the p21 protein shows pleiotropic effects on cell growth and apoptosis in both malignant and non-malignant cells and tissue (13–16). In the nucleus, the p21 protein can function as a tumor suppressor by acting as a cdk2 inhibitor thereby initiating cell cycle arrest in response to DNA damage (17), and also as an oncogene by increasing the assembly of CDK4/6 and cyclin D that initiates entry into the S-phase (18, 19). The cytoplasmic localization of p21 is mainly driven by Akt mediated phosphorylation on Thr145 (p-p21) (20) and when located in the cytoplasm, p-p21 prevents apoptosis (21, 22), further establishing its role as an oncogene.

Few small molecule inhibitors of p21, with the aim to inhibit p21 for potential cancer therapy, have been reported so far. These include butyrolactone I (23), LLW10 (24), sorafenib (25), and the recently developed small molecular inhibitor UC2288, which was developed through modification of sorafenib (26). UC2288, which functions independently of p53, is a more specific p21 inhibitor than sorafenib and attenuates the p21 protein at the level of transcription or post-transcription. In addition, UC2288 markedly decreases cytosolic p21 protein levels, thereby inhibiting the ability of p21 to convey an anti-apoptotic function (26).

The possible role of p21 as an oncogene has previously not been studied in high-risk NB. Therefore, we have here mapped the endogenous expression of p21, both un-phosphorylated and phosphorylated, in a panel of eight high-risk NB cell lines and one NB mouse model. The effect of UC2288, either alone or in combination with cisplatin, was examined to investigate how functional modulation of p21 and p-p21 may alter NB cell viability. Here we report that increased cisplatin resistance was correlated to p-p21, and that combination treatment with UC2288 and cisplatin considerably decreased tumour cell viability. These results suggest an important role for p21 and p-p21 in preventing cisplatin-induced cell death in high-risk NB.

## Material and methods

### Cell Culturing

BE(2)-C and Kelly were obtained from ATCC (Manassas, VA, USA). SK-N-SH, SH-SY5Y, IMR-32, SK-N-AS, SK-N-FI, SK-N-DZ, and MRC5 were a kind gift from professor Per Kogner at Karolinska Institute. HL-60 was a kind gift from Dr. Mohammad Hojjat Farsangi at Karolinska Institute. Eight high-risk NB cell lines were used, where three were *TP53* wild type (wt) and five were *TP53* mutated (mut) in order to compare between *TP53* dependent and independent expression of p21. The non-tumourigenic fibroblast cell line MRC5 and the acute promyelocytic leukemia cell line HL-60 were included as controls (**Supplementary Table S1**). All cells were cultured in RPMI 1640 medium supplemented with 10% fetal bovine serum, 2 mM L-glutamine and 1% penicillin/streptomycin (all from Life Technologies Inc, Thermo Fisher Scientific, Stockholm, Sweden). Cells were incubated at 37°C with 5% CO2 and high humidity.

### Mouse model

The transgenic *TH-MYCN* animals were obtained from the Mouse Model of Human Cancer Consortium Repository and kept on a 129X1/SvJ background (27). Tumours from the transgenic neuroblastoma *TH-MYCN* model are morphologically and phenotypically similar to human high-risk NB. All mice were housed in standard cages in a temperature- and humidity-controlled room with 12-hour light/12-hour dark cycles and ad libitum access to water and food. Genotyping was performed from ear tissue biopsies using qPCR with specific probes designed for wild-type and the *MYCN* transgene (Transnetyx, Cordova, TN, USA). We here used tumours from hemizygous mice (*MYCN* ^+/−^) for immunofluorescent staining for p21 and p-p21 according to the immunofluorescent protocol description.

### Western blot analysis

Twenty-four hours after plating the protein from the NB cell lines, the MRC5, and the HL-60 cell lines were extracted for the baseline data. The protein extraction was done by using RIPA buffer (Thermo Fisher Scientific, Stockholm, Sweden) supplemented with a protease and phosphatase inhibitor (Thermo Fisher Scientific, Stockholm, Sweden) according to the manufacturer’s recommendations, separated electrophoretically and blotted on a nitrocellulose membrane (Bio-Rad, California, USA) using standard procedures. All samples were loaded at 20 µg for equal loading. The p21 protein was detected using an anti-p21 antibody (Cell Signaling, #2947, 1:500) or an anti-p-p21 (T145) antibody (Abcam, #ab47300, 1:250), and Cleaved-Caspase3 (C-Casp3) was detected using an anti-C-Casp3 antibody (Cell Signaling, #9661, 1:1000). GAPDH was stained with anti-GAPDH antibody (Thermo Fisher, #39-8600, 1:4000). Protein bands were visualized with secondary antibodies diluted 1:15,000 (IRDye680 and IRDye800, LI-COR, Nebraska USA) using a ChemiDoc MP imaging system (Bio-Rad, California, USA) or a LI-COR imaging system (LI-COR Biosciences UK Ltd). Analysis of band intensity was performed using ImageJ. All experiments were done in triplicate or quadruplicate, except for the HL-60 experiments which were done in duplicates.

### Real time quantitative polymerase chain reaction

Each cell line in logarithmic growth was plated onto petri dishes and incubated for 24 hours before samples for RT-qPCR were harvested. mRNA was harvested according to the RNeasy Mini Kit protocol (Qiagen, Hilden, Germany). ß-mercaptoethanol was added at a volume of 10 µl/ml of Buffer RLT. mRNA concentration was measured using Nanodrop™ (Thermo Fisher Scientific, Stockholm, Sweden). Reverse transcription of mRNA into cDNA was performed according to the Quantitect^®^ Reverse Transcription manufacturer protocol (Qiagen, Hilden, Germany). RT-qPCR was performed following QuantiText^®^ SYBR^®^ Green PCR manufacturer protocol (Qiagen, Hilden, Germany). The primer sequences used can be found in **Table 1**. HPRTI was used as a housekeeping gene for the NB cell lines as this has been found to be one of the most stably expressed genes in NB (28), while GAPDH was used as a housekeeping gene for the MRC5 and the HL-60 cell lines. HL-60 was used as the calibrator sample to calculate relative expression. All experiments were done in quadruplicates.

**Table 1:** Primer sequences for HPRTI (housekeeping gene for NB cell lines), GAPDH (housekeeping gene for MRC5 and HL-60) and p21, respectively.

Target gene	Primer sequence forward	Primer sequence reverse
HPRTI	TGACACTGGCAAAACAATGCA	GGTCCTTTTCACCAGCAAGCT
GAPDH	GAAGGTGAAGGTCGGAGTC	GAAGATGGTGATGGGATTTC
p21	AGGTGGACCTGGAGACTCTCAG	TCCTCTTGGAGAAGATCAGCCG

The primer annealing temperature used was 60°C.

### Immunofluorescence

Each cell line in logarithmic growth was plated onto round 13 mm #1 coverslips (VWR, Stockholm, Sweden) in petri dishes and incubated for 24 hours before being fixated for 15 minutes using 4% paraformaldehyde (Merck, Molsheim, France). TNB buffer (0.5 g blocking reagent [PerkinElmer, Stockholm, Sweden] to 100 mL TBS buffer [Tris/NaCl pH 7.4]) block was added for 30 min at room temperature. Primary antibody diluted in 0.3% Triton X-100, 0.1% NaN_3_ in 1xPBS was added and the cells were incubated overnight at +4°C. The primary antibody was then removed, and cells were washed 3x5 min with 1xPBS before the secondary antibody, diluted in TNB buffer, was added. The cells were kept at room temperature for 2 hours before the secondary antibody was removed and the cover slips were washed 3x5 min with 1xPBS. The cells were mounted with Prolong Gold antifade with DAPI (Thermo Fisher Scientific, Stockholm, Sweden) to stain the cell nuclei. A Metafer^®^ Slide Scanning Platform (version 3.13.4, Metasystems, Heidelberg, Germany) was used to analyse the cells. The spatial localization of either the p21 or p-p21 expression was done by optical inspection by two independent observers. The mean intensity (mean fluorescence in arbitrary units per cell ± SD) was provided by the Metafersystem software and the low, intermediate, or high expression levels were based on equal range distributions of the values. Following the protocol for immunofluorescence, a triple staining was performed on all eight cell lines using antibodies for p21, the DNA damage marker phosphorylated ataxia-telangiectasia mutated (pATM), and the replication marker Ki67. All experiments were done in triplicate. Antibodies used can be found in **Table 2**.

**Table 2:** Antibodies used for immunofluorescence.

Antibody	Code	Company	Dilution
Anti-p21^Waf1/Cip1^ (12D1)(rabbit)	2947	Cell Signaling Technologies	1:1000
Anti-p21^Waf1/Cip1^ (phospho T145) (rabbit)	ab47300	Abcam	1:1000
Anti-ATM (phospho S1981) (mouse)	ab36810	Abcam	1:500
Anti-Ki67 (chicken)	ab254123	Abcam	1:1000
Anti-C-Casp3 (Asp175) (rabbit)	9661	Cell Signaling Technologies	1:1000
Alexa flour 594 Anti-Rabbit IgG	711-585-152	Jackson ImmunoResearch	1:800
Cy2 Anti-Mouse IgG	115-225-146	Jackson ImmunoResearch	1:400
Alexa Fluor 647 Anti-Chicken IgY (IgG)	703-605-155	Jackson ImmunoResearch	1:400

### Inhibition study

Twenty-four hours after plating of Kelly and SH-SY5Y, UC2288 at a dose of 10 µM (Merck, Molsheim, France) diluted in dimethyl sulfoxide (DMSO) was added for 2 hours, 4 hours or 6 hours before the cells were fixated for 15 min with 4% paraformaldehyde and treated according to the protocol for immunofluorescence, or collected for protein or mRNA according to the protocol for either Western blot or RT-qPCR. UC2288 was added at a concentration of 10 µM based on the literature (29). All experiments were done in triplicate or quadruplicates. The BE(2)-C cell line was treatment for 24 hours with mock (DMSO), cisplatin at a dose of IC_50_ (Accord Healthcare Limited, Middlesex, UK), UC2288 at a dose of 10 µM or a combination of cisplatin and UC2288 followed by fixation for 15 min with 4% paraformaldehyde and treated according to the protocol for immunofluorescence or collected for protein according to the protocol for Western blot. All experiments were done in quadruplicates.

### Cell viability assay

Dose–response curves for cell viability were obtained after treatment for 72 hours with either UC2288 or cisplatin, in eight NB cell lines and one fibroblast cell line, MRC5, which was used as a non-tumourigenic control for drug toxicity. UC2288 was added as a single agent at concentrations of 50 µM, 10 µM, 5 µM, 1 µM, 0.5 µM, 0.1 µM, 0.05 µM or 0.01 µM and cisplatin at concentrations of 100 µM, 50 µM, 10 µM, 5 µM, 1 µM, 0.5 µM, 0.1 µM or 0.05 µM. For the combination treatments, cisplatin was added at concentrations of 100 µM, 50 µM, 10 µM, 5 µM, 1 µM, 0.5 µM, 0.1 µM or 0.05 µM either alone or in combination with UC2288 at a concentration of either 10 µM or 1 µM. The plates were incubated at 37°C, 5% CO_2_. Seventy-two hours after treatment, 3-(4,5-dimethylthiazol-2-yl)-5-(3-carboxymethoxyphenyl)-2-(4-sulfophenyl)-2H-tetrazolium (MTS) assay was performed where 20 µl CellTiter 96^®^ Aqueous One Solution Assay (Promega, Stockholm, Sweden) was added to each well. Plates were incubated for 2.5 hours and metabolic activity was analysed in the microplate reader FLUOstar Omega (BMG LABTECH, Ortenberg, Germany) by measuring absorbance at 490 nm and 690 nm. All treatments were done in triplicate.

### Confluency assay

SK-N-FI (20 × 10^3^ cells), Kelly (10 × 10^3^ cells), SK-N-DZ (10 × 10^3^ cells) and BE(2)-C (5 × 10^3^ cells) were seeded in 200 µl medium/well in a 96 well plate, and the edges were filled with medium to avoid edge effects. Twenty-four hours after seeding each cell line was treated with the corresponding IC_50_ value for cisplatin and 10 µM UC2288 for 48 hours. DMSO (mock) was used as negative controls. The plates were scanned by an IncuCyte S3 Live^®^ Cell Analysis System (Essen Bioscience, Welwyn Garden City, UK) 48 hours post treatment followed by medium change. The plates were thereafter scanned followed by medium change every 96 hours for a total of 30 days. The proliferation was determined by measuring the cell confluency. Once the cell lines reached 100% confluency, they were maintained for the duration of the experiment. Each treatment was done in 5-10 replicates and the data are presented as mean ± Standard Error of the Mean (SEM).

### Combination index analysis

To estimate the effects of the combination treatments the median-effect method of Chou (Chou-Talalay method) (30) was applied to compute combination index (CI) with the ComboSyn software (http://www.combosyn.com; ComboSyn, Inc). In general, a CI of <1 is considered a positive and a CI of >1 a negative combined effect. More specifically, CI<0.70 was defined as synergy, CI>1.45 as antagonism, and values in between as additive effects, according to the recommendations of the ComboSyn software.

### Statistical analysis

Following the MTS-assay, the data was analysed to obtain the IC_50_ value for each of the single treatments or for the combination treatment. The data was logarithmically transformed and normalized against 0% (no cell viability) and 100% (maximum cell viability) controls for the combination treatments. For determining IC_50_ values for the single treatments, the nonlinear regression analysis method log(inhibitor) vs normalized response was used. For determining IC_50_ values for combination treatment, the nonlinear regression analysis log(inhibitor) vs normalized response – variable slope was used. Linear regression analysis with Pearson’s correlation coefficient was used to investigate a possible relationship between the IC_50_ values of UC2288 and cisplatin, and between the fraction of p21 positive cells with either UC2288 or cisplatin. All analyses were performed in Graphpad Prism (version 9.3.1). Western blot data are presented as mean ± standard deviation (SD). Immunofluorescence data were analysed in RStudio and the percentage of positive cells followed by the mean ± standard deviation (SD) were calculated for each combination. Data was analysed using Student’s t-test or one-way ANOVA with Tukey *post hoc* test, as indicated in the text. Graphpad Prism (version 9.1.0) was used for generation of graphs.

## Results

### Investigation of endogenous p21 and p-p21 expression

**Figure 1:**
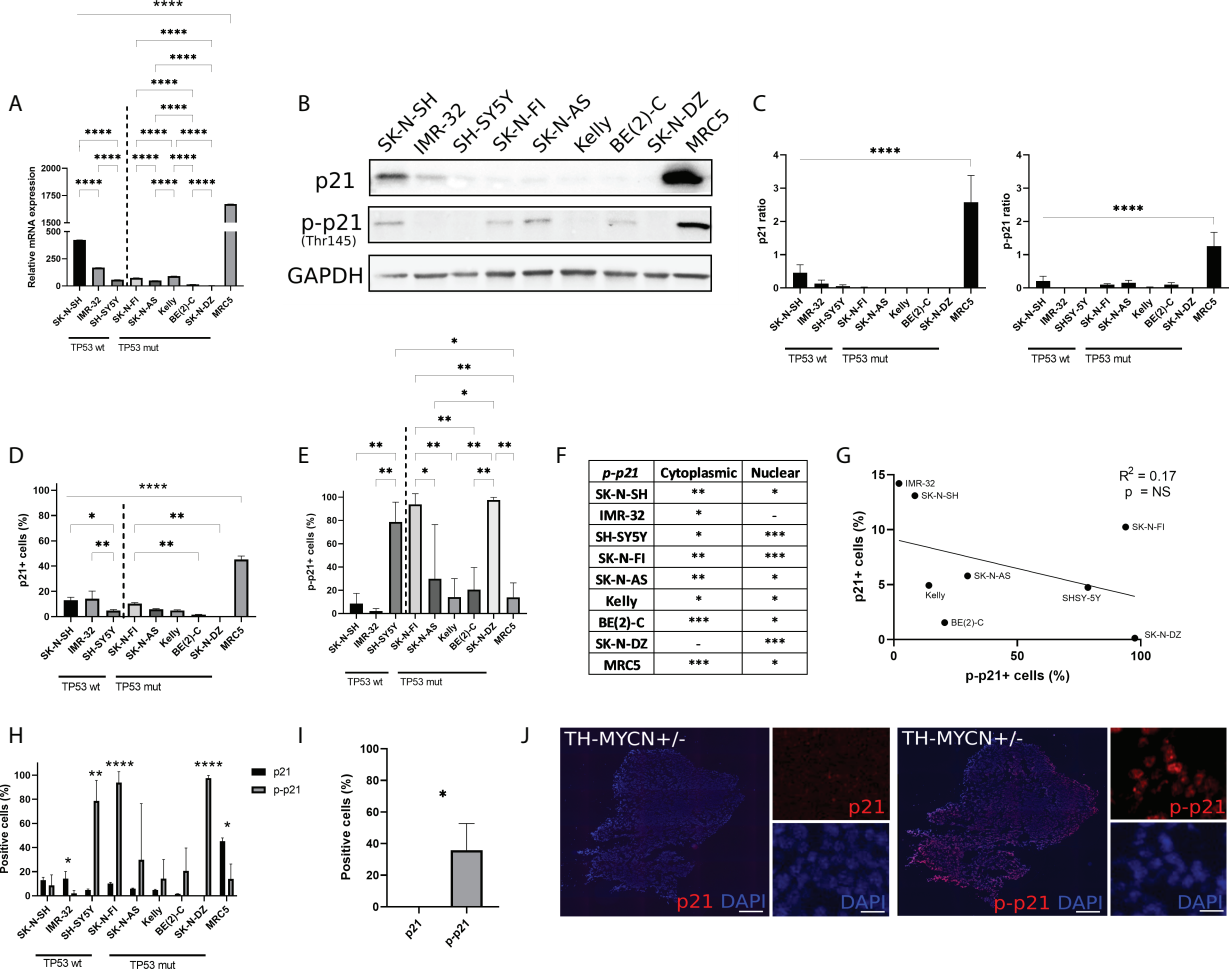
Endogenous p21 and p-p21 expression. **(A)** Relative expression of p21 mRNA (CDKN1A), normalized to HL-60. The MRC5 cell line showed the highest endogenous expression compared to the remaining cell lines. Among the NB cell lines the *TP53* wt cell line SK-N-SH showed the highest endogenous expression compared to the remaining cell lines. * = p<0.05, ** = p<0.01, *** = p<0.001, **** = p<0.0001, not significant p>0.05. One-way ANOVA with Tukey *post hoc* test. Mean ± SEM, n=4. **(B)** Western blot analysis showing protein expression of p21 (21 kDa), p-p21 (Thr145) (32 kDa), and loading control GAPDH (37 kDa) in NB cell lines and MRC5. **(C)** Western blot analysis showed the highest endogenous expression of p21 or p-p21 in MRC5 compared to the remaining cell lines. **** = p<0.0001, not significant p>0.05. One-way ANOVA with Tukey *post hoc* test. Mean ± SD, n=3-4. **(D)** Fraction of p21 expressing cells in each cell line. The highest fraction of p21 positive cells was observed in the MRC5 cell lines. Among the NB cell lines, the *TP53* wt cell lines showed generally a higher fraction of cells positive for p21 compared to the *TP53* mut cell lines. * = p<0.05, ** = p<0.01, **** = p<0.0001, not significant p>0.05. One-way ANOVA with Tukey *post hoc* test. Mean ± SD, n=3. **(E)** Fraction of p-p21 (Thr145) expressing cells in each cell line. Three cell lines, SH-SY5Y, SK-N-FI, and SK-N-DZ showed the highest fraction of p-p21 positive cells. * = p<0.05, ** = p<0.01, not significant p>0.05. One-way ANOVA with Tukey *post hoc* test. Mean ± SD, n=3. **(F)** Investigation of spatial localization of the p-p21 protein. The BE(2)-C and MRC5 cell lines showed highest cytoplasmic p-p21 expression, whereas SH-SY5Y, SK-N-FI, and SK-N-DZ showed the highest nuclear p-p21 expression. - = no expression, * = low expression, ** = moderate expression, *** = high expression. n=3. **(G)** Fraction p21 versus p-p21 positive cells and the correlation between the two. A trend in the correlation was observed, however it was not significant. Linear regression with Pearson’s correlation coefficient, NS= not significant p>0.05. n=3. **(H)** Comparison between the fraction of p21 and p-p21 expressing cells in each cell line. IMR-32 and MRC5 had a lower fraction of p-p21 positive cells compared to p21 positive cells, whereas SH-SY5Y, SK-N-FI, and SK-N-DZ had a higher fraction of p-p21 positive cells compared to p21 positive cells. * = p<0.05, ** = p<0.01, **** = p<0.0001, not significant p>0.05. Student´s t-test. Mean ± SD, n=3. **(I)** The *TH-MYCN* heterozygote mouse model did not show p21 positive cells, whereas 35.9% ± 16.8 of tumour cells where positive for p-p21. * = p<0.05. Student´s t-test. n=3. **(J)** representative pictures of p21 and p-p21 staining on tumour tissue in the *TH-MYCN* heterozygote mouse model. Scale bar 200 µm and 20 µm, respectively.

Real time-qPCR was used in order to determine the baseline (i.e. without prior treatment) mRNA expression level of p21 (CDKN1A) within eight high-risk NB cell lines and one non-tumourigenic fibroblast cell line, MRC5. The MRC5 cell line had the highest p21 mRNA expression compared to the remaining cell lines (p<0.0001) (**Figure 1A**). Comparing the expression of p21 mRNA within *TP53* wt cell lines or *TP53* mut cell lines showed that among the *TP53* wt cell lines SK-N-SH had the highest p21 mRNA expression followed by IMR-32 and among the *TP53* mut cell lines SK-N-FI and Kelly showed the highest p21 mRNA expression (**Figure 1A**). These results were further investigated using Western blot for analysis of protein expression. MRC5 showed the highest expression of p21 among the tested cell lines (p<0.001) (**Figures 1B**). The NB cell lines had low expression of p21, with the expression being below detection levels in two cell lines, BE(2)-C and SK-N-DZ (**Figures 1B, C**). Investigation of p-p21 showed that the expression was highest in MRC5 among the tested cell lines (p<0.001) (**Figures 1B, C**). The NB cell lines had low expression of p-p21, with the expression being below detection levels in four cell lines, IMR-32, SH-SY5Y, Kelly, and SK-N-DZ (**Figures 1B, C)**.

In order to investigate the possible occurrence of p21 and p-p21 expressing cells that might not be detected in bulk analysis we used the Metafer^®^ slide scanning system, which facilitates high-throughput quantitative immunofluorescence microscopy. This allowed the determination of p21 and p-p21 protein expression in single cells. In line with the Western blot data, the fibroblast cell line, MRC5 showed the highest fraction of p21 positive cells (45.3% ± 2.7, p<0.001) among all the tested cell lines (**Figure 1D**). However, unlike the Western blot data, p21 expressing cells were detected in all the NB cell lines. This is in line with the RT-qPCR data (**Figure 1A**). Moreover, comparing the expression of p21 within *TP53* wt cell lines or *TP53* mut cell lines showed that the fraction of p21 positive cells was higher in the *TP53* wt cell lines SK-N-SH (13.1% ± 2.2, p<0.05) and IMR-32 (14.2% ± 6.0, p<0.01), compared to SH-SY5Y (4.7% ± 0.9). In the *TP53* mut cell lines, the fraction of p21 positive cells was generally lower with SK-N-FI (10.2% ± 0.9, p<0.01) having a significantly higher fraction of positive cells compare to BE(2)-C (1.5% ± 0.3) and SK-N-DZ (0.1% ± 0.1). Cells positive for p21 were also detected in SK-N-AS (5.7% ± 0.6) and Kelly (4.9% ± 0.5) (**Figure 1D**). Furthermore, the p21 protein was predominantly localized in the nuclear compartment, with no detectable staining of cytoplasmic p21, in all the tested cell lines (**Supplementary Figure S1A**).

Investigation of the p-p21 expression showed that the fibroblast cell line MRC5 had a low fraction of p-p21 positive cells (14.0% ± 12.3, p<0.05) compared to the three cell lines with the highest expression, SH-SY5Y (78.6% ± 17), SK-N-FI (93.9% ± 9.1), and SK-N-DZ (97.6% ± 2.2) (**Figure 1E**). Moreover, SH-SY5Y (78.6% ± 17, p<0.01) had the highest fraction of positive cells among the *TP53* wt cell lines SK-N-SH (8.6% ± 8.8) and IMR-32 (2.1% ± 2.2). Among the *TP53* mut cell lines, SK-N-FI (93.9% ± 9.1) and SK-N-DZ (97.6% ± 2.2) had significantly higher fractions of p-p21 positive cells compared to SK-N-AS, Kelly, and BE(2)-C cell lines which had moderate expression (29.9% ± 46.6, 14.2% ± 15.9, and 20.6% ± 19.0, respectively) (**Figure 1E**).

Unlike the p21 expression p-p21 was observed in both the cytoplasm and in the nucleus. Quantification of p-p21 expression showed that the p-p21 protein was predominantly localized in the cytoplasm for MRC5 and BE(2)-C, which displayed high expression. Moderate cytoplasmic expression was observed in SK-N-SH, SK-N-FI, and SK-N-AS, followed by low or no detectable cytoplasmic staining in IMR-32, SH-SY5Y, Kelly, and SK-N-DZ (**Figure 1F**; **Supplementary Figure S1B**). The three cell lines displaying the highest fraction of p-p21 positive cells, SH-SY5Y, SK-N-FI, and SK-N-DZ, all had predominant nuclear expression of the protein (**Figure 1F**). However, these cells did not show high expression of p-p21 when investigated with Western blot, instead the results from the Western blot correlated to the cytoplasmic expression of p-p21 where cells with high or moderate expression showed a band (**Figure 1B, F**). There seems therefore to be a discrepancy between the p-p21 antibody when used either with Western blot or with immunofluorescence where Western blot mainly detects cytoplasmic expression and immunofluorescence detects both cytoplasmic and nuclear expression. In order to investigate the specificity of the p21 and the p-p21 antibodies the HL-60 cell line was used since this cell line has the lowest expression of p21 mRNA among a panel of 69 tested cell lines (https://www.proteinatlas.org/ENSG00000124762-CDKN1A/cell+line). No p21 or p-p21 staining was detected in the HL-60 cell line when investigated with Western blot and immunofluorescence validating the specificity of the antibodies (**Supplementary Figure S2**).

When further investigating the expression of p-p21, a possible trend towards a reverse correlation to p21 was observed, however this was not significant (**Figure 1G**). Comparing the expression of p21 and p-p21 among the tested cell lines, two cell lines, IMR-32 (14.2% ± 6.0 > 2.1% ± 2.2, p<0.05) and MRC5 (45.3% ± 2.7 > 14.0% ± 12.3, p<0.05) had a lower fraction of p-p21 positive cells, whereas SH-SY5Y (4.7% ± 0.9 < 78.6% ± 17.1, p<0.01), SK-N-FI (10.2% ± 0.9 < 93.7% ± 9.1, p<0.0001), and SK-N-DZ (0.1% ± 0.1 < 97.6% ± 2.2, p<0.0001) had a higher fraction of p-p21 positive cells (**Figure 1H**).

The protein expression of p21 and p-p21 was also investigated in tumour material from the *TH-MYCN* heterozygote mouse model. The human MYCN cDNA is placed downstream of the tyrosine hydroxylase promoter (Th-MYCN), and the mice spontaneously develop NB at 5.6–19 weeks of age (27, 31). In this material, no cells were detected positive for p21 whereas a significantly higher fraction of tumour cells showed expression of p-p21 (0.0% ± 0.0 < 35.9% ± 16.8, p<0.05) (**Figure 1I**, **J**).

Taken together, the RT-qPCR, Western blot, and immunofluorescence data showed that p21 is expressed, in both an un-phosphorylated and a phosphorylated form, heterogeneously across all eight NB cell lines and in the *TH-MYCN* mouse model. MRC5 showed the highest expression of p21 among all the tested cell lines followed by the *TP53* wt cell line SK-N-SH. Moreover, three NB cell lines, SH-SY5Y, SK-N-FI, and SK-N-DZ, and the *TH-MYCN* mouse model showed a preference for p-p21 expression over p21.

### Endogenous p21 expression is compatible with replication

**Figure 2:**
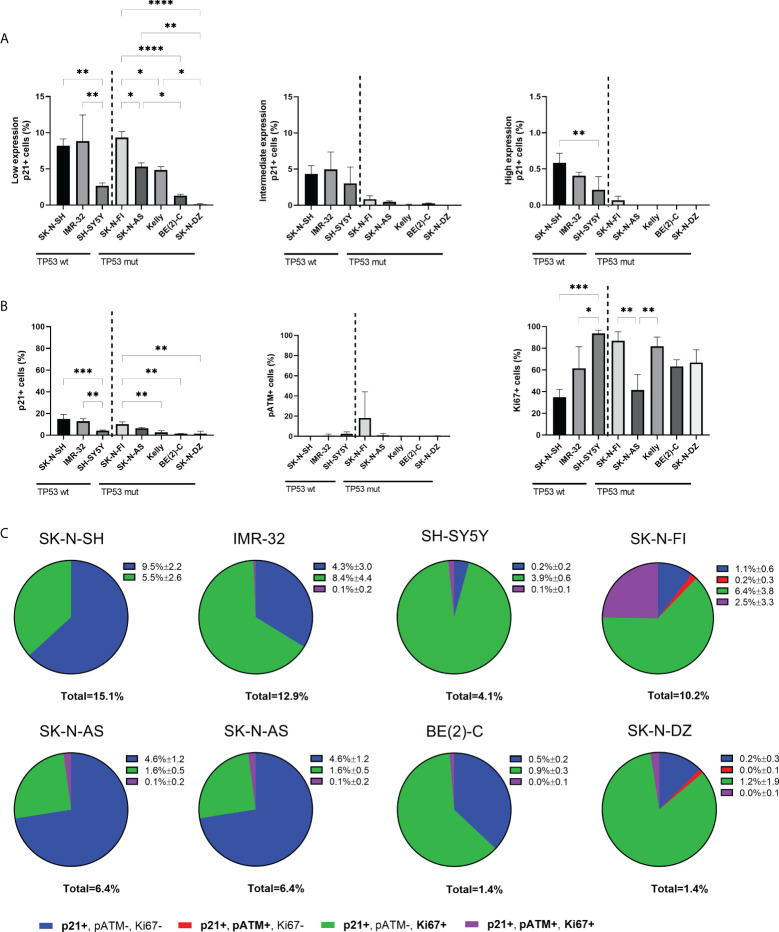
Expression levels of p21 and triple immunofluorescence staining with p21, pATM, and Ki67 in NB cell lines. **(A)** Graphs depict percentage of positive cells for low, intermediate, or high endogenous p21 expression. The *TP53* wt cell lines showed cells with expression levels of p21 in all three ranges, whereas only SK-N-FI had expression levels of p21 in all three ranges among the *TP53* mut cell lines. * = p<0.05, ** = p<0.01, **** = p<0.0001, not significant p>0.05. One-way ANOVA with Tukey *post hoc* test. Mean ± SD, n=3. **(B)** Analyzing the fraction of either p21, pATM or Ki67 single positive cells within all cell lines showed overall low fraction of cells positive for either p21 or pATM and a higher fraction of Ki67 expressing cells. * = p<0.05, ** = p<0.01, *** = p<0.001, not significant p>0.05. One-way ANOVA with Tukey *post hoc* test. Mean ± SD, n=3. **(C)** Pie charts of the four different combinations of p21 expressing cells within each cell line, either single p21 positive, p21 and pATM double positive cells, p21 and Ki67 double positive cells or p21, pATM, and Ki67 triple positive cells. The majority of cell lines showed that there are large subpopulations of replicating cells with p21 expression. Mean ± SD, n=3.

Investigation of p21 expression showed positive nuclear staining in a fraction of cells in all the tested NB cell lines (**Figure 1**). In the nucleus, p21 generally inhibits cyclin-CDK complexes, thus leading to the direct inhibition of cell proliferation (32). However, low expression of p21 has been shown to act as an assembly factor of CDK4/6 and cyclin D thereby aiding initiation of the S-phase (18, 19). Further investigation of the p21 expression in the NB cell lines showed that within the fraction of p21 positive cells, there was heterogeneity in the expression level, with cells expressing low, intermediate, or high levels of p21. Cells with low p21 expression were observed in all the tested cell lines, with SK-N-SH, IMR-32, and SK-N-FI having the highest fraction (8.2% ± 0.9, 8.8% ± 3.6, and 9.3% ± 0.8, respectively). Among the *TP53* wt cell lines, SK-N-SH (8.2% ± 0.9, p<0.01) and IMR-32 (8.8% ± 3.6, p<0.01) showed higher fraction of positive cells compared to SH-SY5Y (2.7% ± 0.4). Among the *TP53* mut cell lines SK-N-FI (9.3% ± 0.8) had the highest fraction of cells expressing low levels of p21 followed by SK-N-AS (5.3% ± 0.5), Kelly (4.8% ± 0.5), BE(2)-C (1.2% ± 0.2), and SK-N-DZ (0.1% ± 0.1) (**Figure 2A**). Cells with intermediate p21 expression were observed in all cell lines except SK-N-DZ. Moreover the *TP53* wt cell lines, SK-N-SH, IMR-32, and SH-SY5Y had the highest fraction of p21 positive cells with intermediate expression among all the tested cell lines. However, there was no significant difference within either the *TP53* wt cell lines or the *TP53* mut cell lines (**Figure 2A**). Cells with high p21 expression were observed in all three *TP53* wt cell lines, with SK-N-SH (0.6% ± 0.1, p<0.01) having a significantly higher fraction of positive cells compared to SH-SY5Y (0.2% ± 0.2). There was only one *TP53* mut cell line with cells positive for high p21 expression, SK-N-FI (0.1% ± 0.1). The remaining *TP53* mut cell lines SK-N-AS, Kelly, BE(2)-C, and SK-N-DZ showed no cells with high p21 expression (**Figure 2A**). Representative picture of low, intermediate, and high p21 expressing SK-N-SH cells are shown in **Supplementary Figure S3A**.

In order to further investigate whether endogenous p21 expression is compatible with proliferation, a triple staining was performed in all eight cell lines, using antibodies binding to p21, together with a downstream effector of cell cycle arrest, phosphorylated ataxia-telangiectasia mutated (pATM), and the proliferation marker Ki67. First, each marker was analyzed on its own with the fraction of p21 positive cells being higher in *TP53* wt cell lines SK-N-SH and IMR-32, followed by the *TP53* mut cell line SK-N-FI, and lower in the remaining cell lines (**Figure 2B**), similar to findings in **Figure 1**. The fraction of pATM positive cells was generally low in all cell lines, except SK-N-FI which had 18.1% ± 25.9 cells positive for pATM. However, there was no significant difference in the fraction of pATM positive cells between the cell lines (**Figure 2B**). Six cell lines showed high fraction of proliferation, > 60% Ki67 positive cells, consistent with active replication. Two cell lines, SK-N-SH and SK-N-AS had a slightly lower fraction of Ki67 positive cells (34.9% ± 7.1 and 41.4% ± 14.4, respectively) (**Figure 2B**).

Analysis of co-expression of the markers showed that within each cell line, a very small fraction of p21 expressing cells displayed co-expression of p21 and pATM (0-0.2%) (**Figure 2C**), confirming that the endogenous p21 expression is primarily not present in response to the DNA damage pathway. Instead, the majority of p21 expressing cells were double positive for Ki67, with the highest fraction of double positive cells observed in SH-SY5Y (95.1%), followed by SK-N-DZ (85.7%), Kelly (65.4%), IMR-32 (65.1%), BE(2)-C (64.3%), and SK-N-FI (62.7%), whereas SK-N-SH (36.4%) and SK-N-AS (25.0%) had the lowest fraction of p21 positive cells which were replicating (**Figure 2C**). Representative pictures of p21 and Ki67 double positive expressing SK-N-DZ cells are shown in **Supplementary Figure S3B**.

Overall, these results indicate that in all cell lines, except SK-N-SH and SK-N-AS, the majority of un-phosphorylated p21 expressing cells were associated with Ki67 and not DNA damage, indicating active replication.

### Analysis of p21 and p-p21 expression following treatment with cisplatin IC_50_

**Figure 3:**
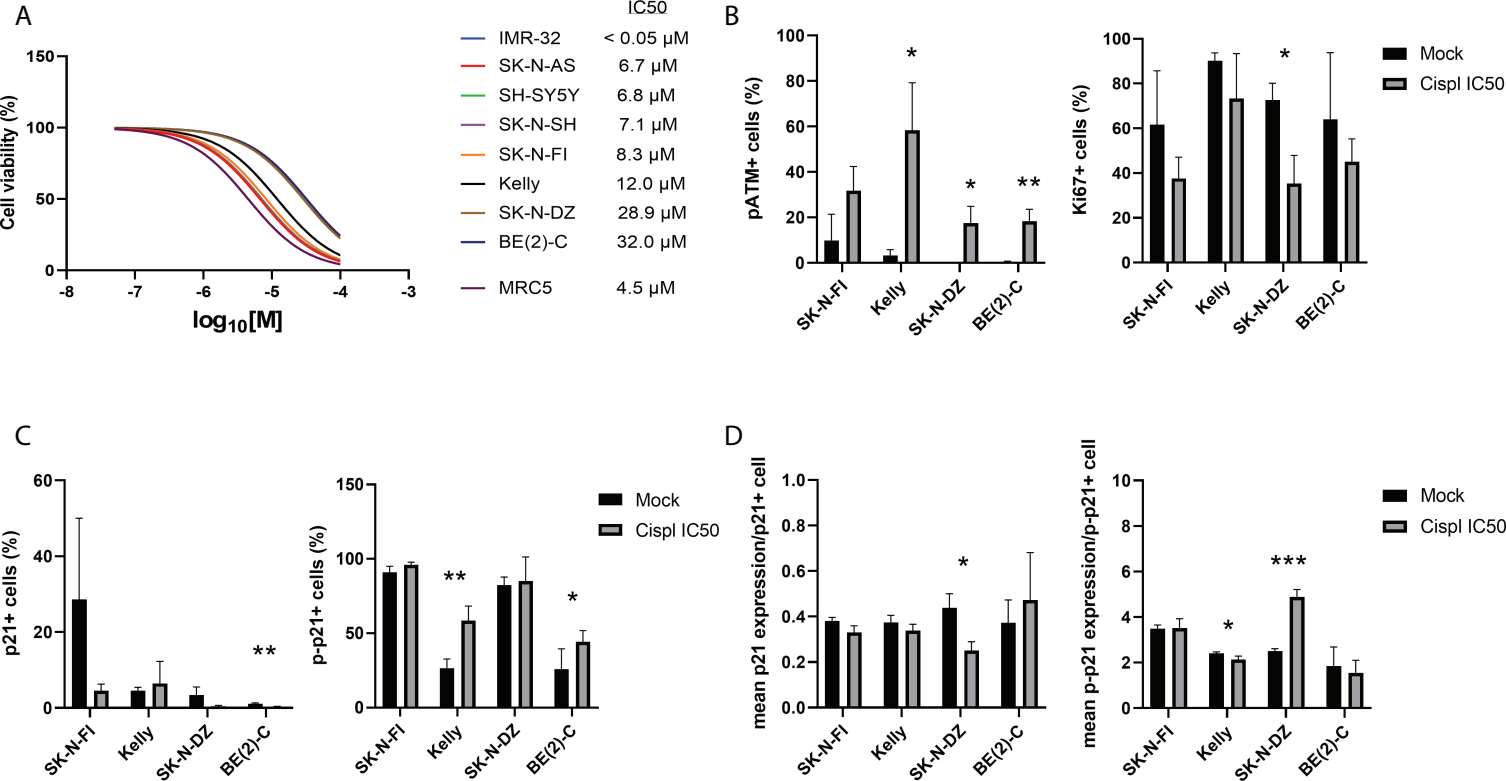
Immunofluorescence staining of p21, p-p21, pATM, and Ki67 in NB cell lines following treatment with cisplatin. **(A)** Dose–response curves for cell viability after 72 hours of cisplatin treatment in eight NB cell lines and the fibroblast cell line, MRC5. Cell viability was assessed using MTS-assay to determine the IC_50_ values of cisplatin as a single agent. Concentration ranges, from 100 µM to 0.05 µM of cisplatin, was added. *TP53* wt cell lines were in general more sensitive compared to the *TP53* mut cell lines. n=3. **(B)** Analyzing the fraction of either pATM or Ki67 single positive cells following treatment with cisplatin for 24 hours at the corresponding IC_50_ concentration for each of the cell lines. The fraction of pATM was increased in three out of four cell lines, and SK-N-DZ showed a reduction in the fraction of Ki67 positive cells following treatment. * = p<0.05, ** = p<0.01, not significant p>0.05, Student´s t-test. Mean ± SD, n=3. **(C)** Analyzing the fraction of either p21 or p-p21 single positive cells following treatment with cisplatin for 24 hours at the corresponding IC_50_ concentration for each of the cell lines. BE(2)-C showed a decrease in the fraction of p21 positive cells, whereas Kelly and BE(2)-C showed an increase in the fraction of p-p21 positive cell. * = p<0.05, ** = p<0.01, not significant p>0.05, Student´s t-test. Mean ± SD, n=3-6. **(D)** Analyzing the mean intensity (mean fluorescence in arbitrary units per cell ± SD) of either p21 or p-p21 single positive cells following treatment with cisplatin for 24 hours at the corresponding IC_50_ concentration for each of the cell lines. SK-N-DZ showed a decrease in the mean intensity of p21 positive cells, whereas Kelly showed a decrease in the mean intensity of p-p21 positive cells, and SK-N-DZ showed an increase in the mean intensity of p-p21 positive cell. * = p<0.05, *** = p<0.001, not significant p>0.05. Student´s t-test. Mean ± SD, n=3-6.

In order to investigate a possible role of p21 and p-p21 following cisplatin treatment, the sensitivity of each cell line towards cisplatin was investigated. Cisplatin showed a concentration-dependent decrease in cell viability after 72 hours of treatment, with IC_50_ values ranging below the tested range for IMR-32 (<0.05 µM) to 32.0 µM for BE(2)-C (**Figure 3A**). Overall, the *TP53* mut cell lines showed a lower sensitivity compared to the *TP53* wt cell lines. Four *TP53* mut cell lines, SK-N-FI, Kelly, SK-N-DZ, and BE(2)-C, displaying the highest IC_50_ values for cisplatin, were selected for further investigation of the cisplatin response. Each of the four cell lines were treated with the corresponding cisplatin IC_50_ concentration for 24 hours following analysis, using immunofluorescence, of DNA damage (pATM) and proliferation (Ki67) as a predictor of treatment outcome (33). An increase in the fraction of pATM positive cells was observed in three out of the four tested cell lines (Kelly 3.3% ± 2.5 < 58.3% ± 20.9, p<0.05; SK-N-DZ 0.1% ± 0.1 < 17.5% ± 7.4, p<0.05; BE[2]-C 0.4% ± 0.3 < 18.3% ± 5.2, p<0.01), indicating activation of the DNA damage response pathway (**Figure 3B**). For one cell line, SK-N-DZ, the fraction of Ki67 positive cells was reduced (72.6% ± 7.5 > 35.4% ± 12.5, p<0.05), whereas a trend in reduction (not significant) was observed for the remaining cell lines (**Figure 3B**). The reduction in proliferating cells seen in the SK-N-DZ cell line might reflect its relatively short doubling time compared to the other cell lines. Further investigation of p21 and p-p21 expression following cisplatin treatment showed a reduction in the fraction of p21 positive cells in the BE(2)-C cell line (1.1% ± 0.2 > 0.2% ± 0.2, p<0.01), whereas an increase in the fraction of p-p21 positive cells was observed in Kelly (26.6% ± 6.1 < 58.5% ± 9.8, p<0.01) and BE(2)-C (25.9% ± 13.7 < 44.3% ± 7.6, p<0.05) (**Figure 3C**). When investigating the fraction of positive cells for each marker we observed a change in the intensity of the expression. Therefore, we also measured the mean expression (mean fluorescence in arbitrary units per cell ± SD) of either p21 or p-p21. In the SK-N-DZ cell line there was a reduction in the mean expression of p21 (0.44 ± 0.06 > 0.25 ± 0.04, p<0.05), whereas an increase was observed for p-p21 (2.50 ± 0.11 < 4.89 ± 0.33, p<0.001), indicating a shift from the un-phosphorylated to the phosphorylated protein (**Figure 3D**). Moreover, Kelly showed a slight but significant reduction in the mean expression of p-p21 following treatment (2.41 ± 0.07 > 2.14 ± 0.15, p<0.05) (**Figure 3D**).

Taken together, the *TP53* mut cell lines were more resistant to cisplatin treatment compared to the *TP53* wt cell lines. Furthermore, an increase in the fraction of pATM positive cells, indicating activation of the DNA damage pathway, was observed following treatment with cisplatin at the corresponding IC_50_ concentration for three of the four cell lines investigated. Two of the four tested cell lines, SK-N-DZ and BE(2)-C, had a decrease either in the fraction or the mean intensity of p21 positive cells. At the same time, three of the four tested cell lines, all but SK-N-FI, had an increase in either the fraction or the mean intensity of p-p21 positive cells, indicating that there is a preference of p-p21 activation, compared to p21, following cisplatin treatment.

### Determination of sensitivity to the p21 inhibitor UC2288

**Figure 4:**
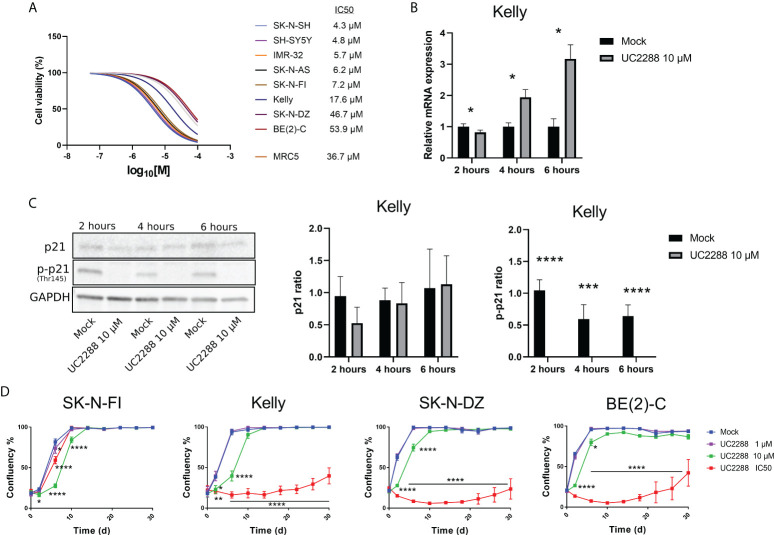
Measuring sensitivity to the p21 inhibitor UC2288. **(A)** Dose–response curves for cell viability after 72 hours of UC2288 treatment in eight NB cell lines and the fibroblast cell line, MRC5. Cell viability was assessed using MTS-assay to determine the IC_50_ values of UC2288 as single agents. Concentration ranges from 50 µM to 0.01 µM of UC2288, were added. n=3. **(B)** p21 inhibition assays performed on Kelly with UC2288 at a concentration of 10 µM at three time points. A reduction in mRNA was observed after 2 hours followed by an increase at 4 hours and 6 hours post-treatment. * = p<0.05, not significant p>0.05, Student´s t-test. Mean ± SD, n=3. **(C)** Western blot analysis showing protein expression of p21 (21 kDa), p-p21 (Thr145) (32 kDa) and loading control GAPDH (37 kDa) in the Kelly cell line following treatment with UC2288 at a concentration of 10 µM at three time points. The expression of p21 was not altered whereas the expression of p-p21 was reduced at all three time points investigated. **** = p<0.0001 not significant p>0.05, Student´s t-test. Mean ± SD, n=4. **(D)** Cultured cells were exposed to the indicated treatment (mock/UC2288) and analysed using live cell imaging with the IncuCyte at the indicated time points. The confluence shows the cellular densities of each cell line measured over 30 days. The lowest tested dose (1 µM) showed delay in the SK-N-FI cell line. Following either IC_50_ concentration or 10 µM all cell lines showed regrowth capacity, albeit after an extended lag period.* = p<0.05, **= p<0.01, *** = p<0.001, not significant p>0.05. Two-way ANOVA with Bonferroni *post hoc* test. Mean ± SEM of 5 experiments.

Most of the tested NB cell lines showed high endogenous expression of p-p21 and/or an activation of p-p21 following cisplatin treatment. In order to investigate the sensitivity of the NB cell lines to the inhibition of p21 and p-p21, a small molecular inhibitor of p21 namely UC2288 was used. Treatment with UC2288 showed a concentration-dependent decrease in cell viability after 72 hours of treatment, with IC_50_ values ranging between 4.3 µM to 53.9 µM (**Figure 4A**).

UC2288 is reported to inhibit p21 transcriptionally and/or post-transcriptionally (26), therefore as a proof of concept, inhibition assays were performed on the *TP53* mut cell line Kelly and the *TP53* wt cell line SH-SY5Y. A concentration of 10 µM of UC2288 was selected, since higher concentrations have been reported to give adverse effects (29). Three time point were selected, 2 hours, 4 hours and 6 hours post-treatment, and p21 mRNA and protein levels were assessed using Western blot and immunofluorescence. In the Kelly cell line, a decrease in the p21 mRNA levels were observed after 2 hours treatment followed by increasing levels after 4 hours and 6 hours treatment (**Figure 4B**), which might suggest stress induction, similar to findings by Taubenberger and colleagues (29). The expression of p21 was not altered when investigated with Western blot, instead UC2288 successfully reduced p-p21 expression following treatment at all three tested time points (p<0.01 or p<0.001) (**Figure 4C**). Moreover, when investigated with immunofluorescence treatment with UC2288 successfully reduced the fraction of p21 positive cells following 4 hours treatment (6.3% ± 2.4 > 1.9% ± 0.5, p<0.05) and the fraction of p-p21 positive cells following 6 hours treatment (10.0% ± 3.2 > 3.5% ± 1.7, p<0.05) (**Supplementary Figure S4A**). The SH-SY5Y cell line showed an increase in p21 mRNA levels after 4 hours and no change in the fraction of either p21 or p-p21 positive cells at any of the tested time points when investigated using immunofluorescence (**Supplementary Figure S4B**). Based on these results, it was confirmed, in the *TP53* mut cell line Kelly, that UC2288 reduces cytoplasmic p-p21 expression.

To determine the long-term effect of UC2288 treatment, regrowth assays were conducted on four *TP53* mut cell lines. Two of these, SK-N-FI and Kelly, showed higher sensitivity to UC2288 compared to the remaining two, SK-N-DZ and BE(2)-C. Regrowth (confluency) was mapped during a period of 30 days following a pulse treatment for 48 hours of mock or UC2288 at three different concentrations: 1 µM, 10 µM or the corresponding IC_50_ value for each cell line. SK-N-FI showed the highest sensitivity to the treatment, with a delay in growth at all three tested concentrations of UC2288 (**Figure 4D**). Kelly, SK-N-DZ, and BE(2)-C showed a delay in growth at a concentration of 10 µM and a lag period of approximately 26 days following IC_50_ dosing of UC2288 before an increase in confluency (regrowth) was observed (**Figure 4D**).

Taken together, UC2288 showed a concentration-dependent decrease in cell viability with *TP53* wt cells being more sensitive. UC2288 was able to reduce both the p21 and the p-p21 expression in the *TP53* mut cell line Kelly, and a delay in regrowth was observed in all four tested *TP53* mut cell lines, indicating sensitivity to the drug.

### Combination treatment with UC2288 and cisplatin

**Figure 5:**
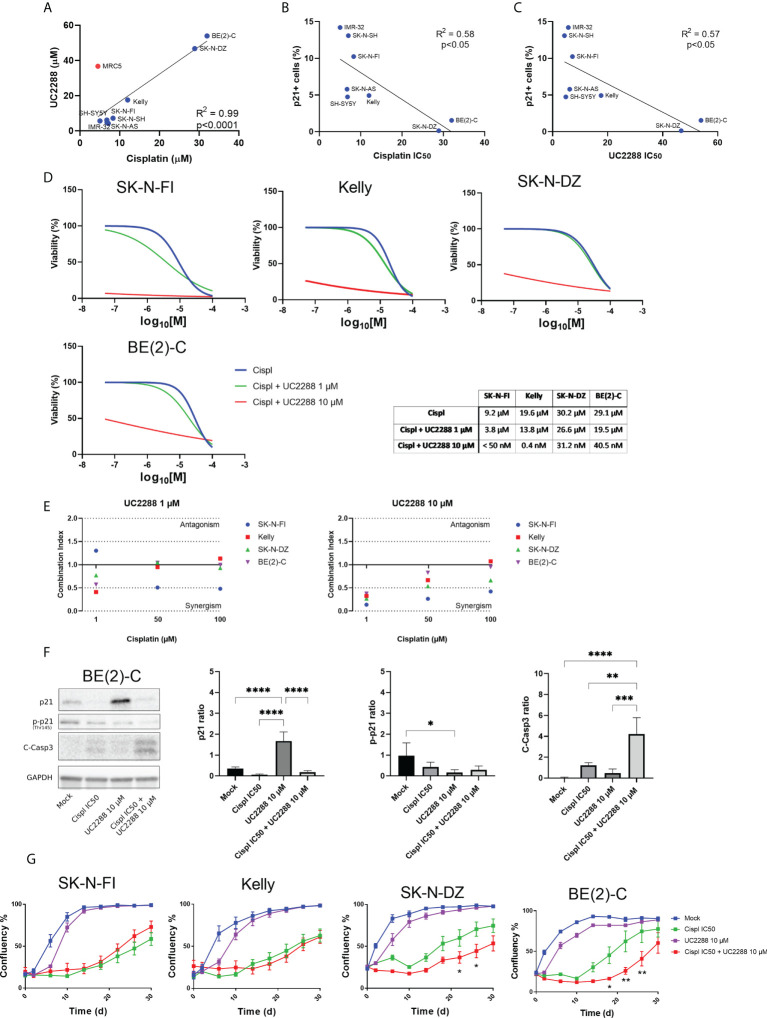
Combination treatment with UC2288 and cisplatin. **(A)** UC2288 versus cisplatin IC_50_ values and the correlation between the two. A high correlation between sensitivity to UC2288 and cisplatin was observed, MRC5 was excluded from the correlation analysis. Overall, SK-N-SH and IMR-32 showed the highest sensitivity to UC2288 and cisplatin whereas SK-N-DZ and BE(2)-C showed most resistance. Linear regression with Pearson’s correlation coefficient, p<0.0001. n=3. **(B)** A reverse correlation was observed for the fraction of p21 positive cells and sensitivity to either cisplatin or **(C)** UC2288. Linear regression with Pearson’s correlation coefficient, p<0.05. n=3. **(D)** Viability assay on four resistant cell lines, Kelly, SK-N-FI, BE(2)-C, and SK-N-DZ, following treatment with cisplatin as a single reagent or in combination with UC2288 (1 µM or 10 µM). A reduction in IC_50_ was observed in all cell lines following combination treatment compared to a single cisplatin treatment. n=3. **(E)** Combination treatments with cisplatin and UC2288 at either 1 µM or 10 µM. Combination index (CI) analysis was calculated in four NB cell lines, CI<0.7 suggests synergy, CI>1.45 antagonism, and 0.7<CI<1.45 additive combinational effects. **(F)** Western blot analysis showing protein expression of p21 (21 kDa), p-p21 (Thr145) (32 kDa), C-Casp3 (19 kDa and 17 kDa), and loading control GAPDH (37 kDa) in the BE(2)-C cell line following 24 hours treatment with Mock, cisplatin at IC_50_, UC2288 at 10 µM, or a combination of cisplatin IC_50_ and UC2288 10 µM. An increase was observed of p21 following UC2288 treatment, a decrease in p-p21 following UC2288 treatment and an increase of C-Casp3 following combination treatment. * = p<0.05, ** = p<0.01, *** = p<0.001, **** = p<0.0001, not significant p>0.05. One-way ANOVA with Tukey *post hoc* test. Mean ± SD, n=4. **(G)** NB cell lines were treated with cisplatin and/or UC2288 and regrowth was measured over 30 days with an IncuCyte (‘confluence’ value indicates the cellular densities). The resistant cell lines, BE(2)-C and SK-N-DZ, showed a reduction in growth following combination treatment with cisplatin and UC2288 compared to a single cisplatin treatment. BE(2)-C showed highest sensitivity for combination of the drugs. * = p<0.05, **= p<0.01. Two-way ANOVA with Bonferroni post tests. Mean ± SEM of 5-10 experiments.

Investigating a possible correlation in resistance between the single agent UC2288 and cisplatin, showed that cell lines with low IC_50_ values for UC2288 (IMR-32, SK-N-SH, SK-N-AS, SH-SY5Y, SK-N-FI, and Kelly) also had low IC_50_ values for cisplatin. On the other hand, BE(2)-C and SK-N-DZ had higher IC_50_ values for both UC2288 and cisplatin, implying that they are more resistant to single agent treatment with these drugs (**Figure 5A**). This is supported by the results showing a reverse correlation between the fraction of p21 positive cells and sensitivity to either cisplatin or UC2288 (**Figures 5B**, **C**). Based on the correlation in resistance between the single agent UC2288 and cisplatin, we investigated whether a combination treatment of the drugs could be a more effective treatment strategy.

The results showed that combination treatment was effective using UC2288 at a dose of 10 µM for both the more sensitive cell lines, Kelly and SK-N-FI, and the more resistant cell lines, BE(2)-C and SK-N-DZ. Due to the huge shift following combination treatment with UC2288 at 10 µM, the IC_50_ values were calculated based on extrapolated values. The cisplatin IC_50_ value shifted from 19.6 µM to 0.4 nM for Kelly and from 9.2 µM to below range (< 50 nM) for SK-N-FI. For BE(2)-C, the cisplatin IC_50_ value shifted from 29.1 µM to 40.4 nM and for SK-N-DZ from 30.2 µM to 31.2 nM (**Figure 5D**). To assess whether a shift would be observed with a lower concentration of UC2288, the same experimental setup was used with UC2288 at a concentration of 1 µM. At 1 µM UC2288, a small shift in the cisplatin IC_50_ value was observed, both in the sensitive and in the resistant cell lines (**Figure 5D**). The combination treatment was also evaluated in the MRC5 cell line, were the cisplatin IC_50_ value shifted from 14.2 µM to below range (< 50 nM) when combined with UC2288 at 10 µM. Moreover, a small shift in the cisplatin IC_50_ value was observed when combined with following UC2288 at 1 µM (**Supplementary Figure S5**). Combination index analysis was also performed to further evaluate a possible positive synergism, the additive effects or antagonism between cisplatin and UC2288. A synergistic effect was observed in all cell lines following combination treatment with cisplatin with UC2288 at 10 µM and at 1 µM (**Figure 5E**). The BE(2)-C cell line was further investigated using Western blot and immunofluorescence following treatment with either mock, cisplatin at IC_50_, UC2288 at 10 µM or a combination of cisplatin IC_50_ and UC2288 10 µM. An increase of p21 was observed with both Western blot and immunofluorescence following UC2288 10 µM treatment (**Figure 5F**; **Supplementary Figure S6**). However, whereas a decrease in p-p21 was observed following UC2288 10 µM treatment with Western blot (**Figure 5F**), this was not observed using immunofluorescence, an increase in the fraction of p-p21 expressing cells was observed following treatment with cisplatin at IC_50_ (**Supplementary Figure S6**). Moreover, an increase of C-Casp3 following combination treatment was observed with both Western blot and immunofluorescence (**Figure 5F**; **Supplementary Figure S6**).

To determine the long-term effect of the combination treatment with UC2288 and cisplatin, regrowth assays were conducted on the four previously selected cell lines. Regrowth (confluency) was mapped during a period of 30 days following a pulse treatment for 48 hours of mock, cisplatin IC_50_, UC2288 10 µM, or combination of cisplatin IC_50_ and UC2288 10 µM for each corresponding cell line. Two cell lines, SK-N-DZ and BE(2)-C, showed delay in growth following combination treatment compared to a single cisplatin treatment (**Figure 5G**).

Taken together, combination treatment with UC2288 and cisplatin reduced viability in both sensitive, Kelly and SK-N-FI, and resistant, BE(2)-C and SK-N-DZ, cell lines, even though the effect was more profound in the sensitive cell lines. Furthermore, combination treatment showed increase in cell death, indicated by C-Casp3, and a delay in regrowth compared to a single treatment with cisplatin in the two resistant cell lines, BE(2)-C and SK-N-DZ.

## Discussion

The p21 protein is highly versatile, with a multifunctional role as both a tumor suppressor and an oncogene. The functional regulation of p21 relies basically on two post-translational modifications: phosphorylation and ubiquitylation (34), where the process of phosphorylation serves to regulate the p21 activity, localization, stability, and degradation. In this study we show in a panel of eight high-risk NB cell lines, endogenous expression of both un-phosphorylated and phosphorylated p21 (Thr145). The expression of p21 and p-p21 was not dependent on functional p53, since both *TP53* wt and *TP53* mut cell lines showed expression. However, there was discrepancies between the expression level of p21 mRNA and the p21 protein, both as un-phosphorylated and phosphorylated protein. This is in concordance with studies showing general poor correlation between expression levels of mRNA and protein (37, 38), a discrepancy which might be cell-type specific and attributed to other levels of regulation between transcript and protein product (39).

Nevertheless, our data support the notion of p21 as an oncogene in NB. The un-phosphorylated p21 protein was expressed in a small fraction of unstressed proliferating cells, in line with the role of p21 as an assembly factor for CDK4/6 complexes during G1 transition (19). However, despite the possible role of p21 in cell cycle progression other drivers i.e. MYCN, CDK4/6, and cyclins, in cell cycle progression most likely reflect the proliferation status of the cell lines. Indeed, both the cell lines SK-N-SH and SK-N-AS, which showed the least fraction of proliferating p21 positive cells also lack amplification of the proto-oncogene MYCN (40).

Whereas the fraction of p21 expressing cells was low among the tested NB cell lines, a higher fraction of cells showed expression of p-p21, displaying heterogeneity in its cellular localization. All of the tested cell lines, except SK-N-DZ, displayed endogenous cytoplasmic localization of p-p21, suggesting that the Thr145 localization driver may be cell-type specific. Similar results have been observed by others where p-p21 was localized both in the cytoplasm and in the nucleus (41, 42). Furthermore, both cytoplasmic and nuclear p-p21 have been suggested to be pro-tumourigenic, where cytoplasmic p-p21 abrogates or downregulates apoptotic responses (21, 32) and nuclear p-p21 causes loss in its ability to interact with the proliferating cell nuclear antigen (PCNA), thereby facilitating proliferation in endothelial cells (42).

Following investigation of sensitivity to cisplatin treatment, the four cell lines displaying the highest resistance were selected for further analysis. Among the selected cell lines all have *TP53* mut and three, SK-N-DZ, Kelly, and BE(2)-C, have *MYCN* amplification. The clinical prevalence of these genetic alterations is seen among 20-30% of NB tumours which have *MYCN* amplification, and *TP53* mut are observed in a high proportion following relapse, indicating a mechanistic relevance in the development of therapy resistance (43). Morover, the p21 protein, either un-phosphorylated or phosphorylated, is rarely inactive following relapse (35, 36), suggesting it might be an attractive therapeutic target. The fraction or the mean intensity of p21 positive cells was either reduced or unaltered in four of the four tested NB cell lines, indicating that p21 might not be a driver of cell cycle arrest in NB following cisplatin treatment. This is similar to previous findings in NB cell lines treated with low concentrations (<0.2µM) of doxorubicin where no induction of p21 was observed (44). However, higher concentrations of doxorubicin (1 µM) have been shown to induce p21 in NB cell lines (45), suggesting that the induction of p21 might be concentration dependent. Moreover, an increase in either the fraction or the mean intensity of p-p21 positive cells was observed in three of the four tested cell lines, Kelly, SK-N-DZ, and BE(2)-C, indicating a preference for the anti-apoptotic function of p-p21 over its cell cycle regulatory function in NB cells, similar to findings in clear-cell carcinomas, testicular cancer, and ovarian cancer treated with cisplatin (11, 12, 46).

In order to sensitize NB cells to cisplatin we used the p21 inhibitor UC2288. First, we investigated the ability of UC2288 to inhibit p21 and/or p-p21 in NB cell lines. In the *TP53* mut cell line Kelly there was an initial reduction in the p21 mRNA expression and a transient reduction in the fraction of p21 positive cells following treatment with UC2288. This indicates that the mechanism by which UC2288 attenuates p21 occurs by means of transcriptional or post-transcriptional regulation and not *via* protein degradation (26). Moreover, a significant downregulation of p-p21 was observed at all three tested time points when analyzed with Western blot and following 6 hours treatment when analyzed with immunofluorescence. The difference between the p-p21 data generated by the two methods most likely reflects the sensitivity of the p-p21 antibody for both cytoplasmic and nuclear p-p21 when applied in immunofluorescence. Indeed, UC2288 has been shown to more selectively target cytoplasmic p-p21 compared to nuclear p21 (26). Furthermore, treating the *TP53* wt cell line SH-SY5Y with UC2288 did not reduce the fraction of p21 or p-p21 positive cells. This most likely reflects the cellular localization of the proteins since SH-SY5Y expresses predominantly nuclear p21 and p-p21. Similar results was also seen following treatment with UC2288 in the BE(2)-C cell line where a reduction of cytoplasmic p-p21 was observed when analyzed with Western blot but not when analyzed with immunofluorescence which detects both cytoplasmic and nuclear p-p21 expression, further validating the ability of UC2288 to inhibit mainly cytoplasmic p-p21. Further treatment of *TP53* mut NB cell lines with UC2288 in combination with cisplatin showed a synergistic effect and a dramatically reduced the IC_50_ value, with an increase in cell death, indicated by C-Casp3 activation, suggesting UC2288´s potential as a drug against chemotherapy resistance (47). Moreover, regrowth was delayed in the two most cisplatin-resistant cell lines, SK-N-DZ and BE(2)-C, when UC2288 was given in combination with cisplatin, further indicating the potential of the p21 inhibitor as a drug targeting cisplatin resistant cells.

Overexpression of cytoplasmic p21 is found in a variety of human cancers, including renal cell carcinoma, breast cancer, pancreatic cancer, testicular cancer, ovarian cancer, cervical cancer, squamous cell carcinomas and prostate cancer (47). In many cases, p21 upregulation correlates positively with poor prognosis, tumour grade, invasiveness and drug-resistance (32, 47). However, in patient derived high-risk NB tumours it was suggested that lower levels of p21 expression could be associated with poorer outcome (48). A reverse correlation was also observed for the fraction of p21 positive cells and sensitivity to either cisplatin or UC2288 in our data, supporting the findings seen in patients. Moreover, our data indicate a general reverse correlation between p21 and p-p21 where cells with low p21, and higher p-p21, are more resistant to treatment (**Figures 1**, **5A-C**). It is therefore important to increase our understanding of the paradoxical function of p21 to effectively design therapeutic strategies. This is of interest since treatment with the UC2288 original construct sorafenib demonstrate inhibition of growth of NB tumours by targeting both NB cells and tumour blood vessels (49), whereas it gave minimal anti-tumour activity in NB patients with relapse and refractory NB (50). The discrepancy between primary and relapse NB tumours treated with sorafenib might be explained by aberrations in signaling pathways regulating p21. Cisplatin has been shown to activate PI3K/Akt in several cancer cell lines (51), suggesting that even in NB chemotherapy induced Akt might drive phosphorylation of p21, thereby shifting the balance towards its oncogenic properties (16). Furthermore, aberrant activation of the PI3K/Akt pathway has been shown to correlate with poor outcome in NB (52, 53). Therefore, targeting both the PI3K/Akt pathway and p21 in combination with chemotherapy might give an even more potent effect. This is also supported by findings where inhibition of the PI3K/Akt signaling pathway was suggested to represent a clinically relevant target for the treatment of high-risk NB patients (54).

In conclusion we demonstrate an important mechanism, dependent on p-p21 expression levels in NB, that mediated resistance to cisplatin. Moreover, we provide a target to overcome resistance, thereby our findings might offer an alternative therapeutic strategy in order to reduce side effects and improve treatment outcome.

## Data availability statement

The original contributions presented in the study are included in the article/**Supplementary Material**. Further inquiries can be directed to the corresponding author.

## Ethics statement

The animal study was reviewed and approved by Stockholm ethics committee for animal research (no. 5163-2019).

## Author contributions

SSF contributed to conceptualization, data curation, methodology, project administration, resources, software, supervision, visualization, and funding acquisition. AS, VH and SSF performed the formal analysis, statistical analysis, investigation, and validation. AS, MW and SSF wrote the first draft of the manuscript. MW and SSF wrote sections of the manuscript. All authors contributed to manuscript revision, read, and approved the submitted version.

## Funding

This research was funded by the Childhood Cancer Foundation grant numbers, TJ2019-0118, PR2019-0101, and PR2021-0037, the Swedish Cancer Society grant number 21-0313 SIA. Eva and Oscar Ahrén Foundation, Stockholm, and Magnus Bergvalls stiftelse grant number 2021-04262.

## Acknowledgments

The authors wish to thank Dr. Per Kogner for NB cell lines and Dr. Mohammad Hojjat Farsangi for the HL-60 cell line. We would also like to thank Dr. Ingrid Lilienthal and Hala Ibrahim Mohammad Habash for scientific discussions and technical assistance.

## Conflict of interest

The authors declare that the research was conducted in the absence of any commercial or financial relationships that could be construed as a potential conflict of interest.

## Publisher’s note

All claims expressed in this article are solely those of the authors and do not necessarily represent those of their affiliated organizations, or those of the publisher, the editors and the reviewers. Any product that may be evaluated in this article, or claim that may be made by its manufacturer, is not guaranteed or endorsed by the publisher.
