# Supplementary material for: The cyclin dependent kinase inhibitor p21Cip1/Waf1 is a therapeutic target in high-risk neuroblastoma
Source: Front Oncol. 2022 Sep 6;12:906194. doi: 10.3389/fonc.2022.906194 (PMC9486206; doi:10.3389/fonc.2022.906194)
Supplement: Supplementary file 1 [file Table_1.docx]

Supplementary Material

**Supplementary Table 1**. List of the eight high-risk NB, one fibroblast, and one acute promyelocytic leukaemia cell lines used.

| Cell line | TP53  status | NMYC status | 11q  status | 17q  status | Alk  Status | Origin |
| --- | --- | --- | --- | --- | --- | --- |
| SK-N-SH | wild type | wild type | wild type | gain | mutated | Metastasis location: Bone marrow  Patient age: 4 years  Gender: Girl |
| SH-SY5Y | wild type | wild type | wild type | gain | mutated | Metastasis location: Bone marrow  Patient age: 4 years  Gender: Girl  Additional info: Cloned subline of SK-N-SH |
| IMR-32 | wild type | amplified | wild type | gain | mutated | Metastasis location: Abdominal  Patient age: 13 months  Gender: Boy |
| SK-N-DZ | mutated | amplified | deletion (q21-qter) | gain | wild type | Metastasis location: Bone marrow  Patient age: 2 years  Gender: Girl |
| Kelly | mutated | amplified | deletion (q23.3-qter) | gain | mutated  F1174L | Metastasis location: brain  Patient age: 1 year  Gender: Girl |
| SK-N-AS | mutated | wild type | deletion (q13.4-qter;  impaired ATM) | gain | wild type | Metastasis location: Bone marrow  Patient age: 6 years  Gender: Girl |
| SK-N-FI | mutated | wild type | wild type | gain | wild type | Metastasis location: Bone marrow  Patient age: 11 years  Gender: Boy |
| BE(2)-C | mutated | amplified | wild type | gain | wild type | Metastasis location: Bone marrow  Patient age: 22 months  Gender: Boy  Additional info: Cloned subline of SK-N-BE(2). Retrieved after repeated chemo and radiotherapy. |
| MRC5 | wild type | wild type | wild type | wild type | wild type | Normal lung tissue (fibroblast) from a 14-week-old male fetus |
| HL-60 | biallelic deletion | cMYC amplified | - | - | - | Acute promyelocytic leukemia from a 36 year-old female |
