# Supplementary material for: The cyclin dependent kinase inhibitor p21Cip1/Waf1 is a therapeutic target in high-risk neuroblastoma
Source: Front Oncol. 2022 Sep 6;12:906194. doi: 10.3389/fonc.2022.906194 (PMC9486206; doi:10.3389/fonc.2022.906194)
Supplement: Supplementary file 2 [file Image_1.pdf]

Supplementary Material

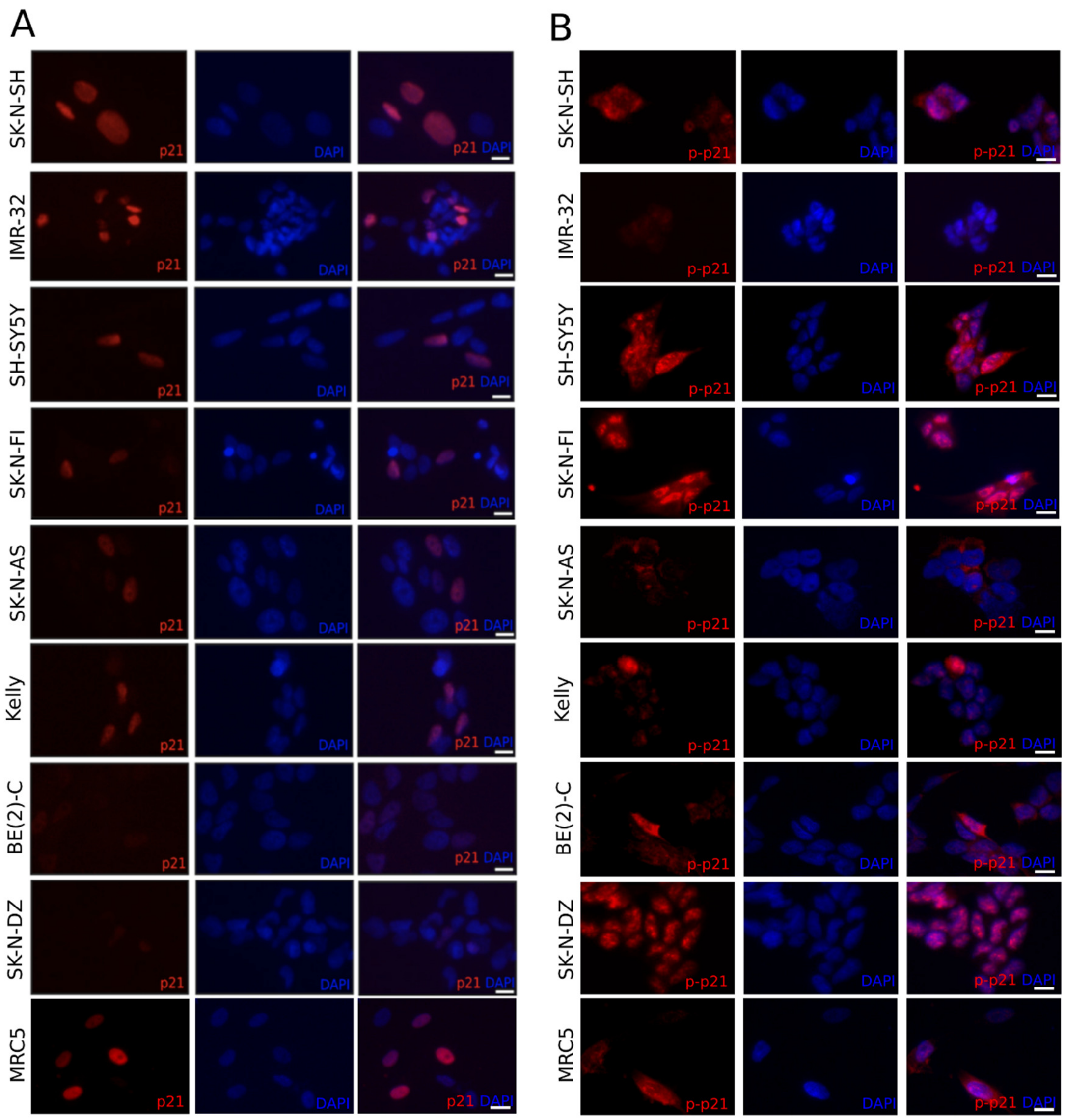

Supplementary Figure 1. Endogenous p21 and p-p21 expression in eight high-risk NB cell lines and one fibroblast cell line, MRC5. (A) Representative images of p21 staining. The p21 protein was predominantly localized in the nuclear compartment in all the tested cell lines. Scale bar = 10 µm. (B) Representative images of p-p21 (Thr145) staining. The p-p21 protein was predominantly localized in the cytoplasm for BE(2)-C and MRC5, in both the nucleus and the cytoplasm for SK-N-SH, SH-SY5Y, SK-N-FI, SK-N-AS, and Kelly, whereas SK-N-DZ showed predominantly nuclear expression, having no detectable cytoplasmic p-p21 staining. Scale bar = 10 µm.
