# Supplementary material for: The cyclin dependent kinase inhibitor p21Cip1/Waf1 is a therapeutic target in high-risk neuroblastoma
Source: Front Oncol. 2022 Sep 6;12:906194. doi: 10.3389/fonc.2022.906194 (PMC9486206; doi:10.3389/fonc.2022.906194)
Supplement: Supplementary file 3 [file Image_2.pdf]

frontiers

Supplementary Material

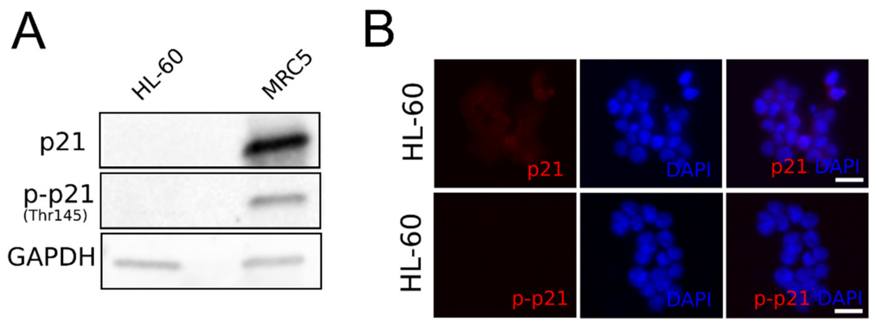

Supplementary Figure 2. Endogenous p21 and p-p21 expression in the acute promyelocytic leukemia cell line, HL-60 and the fibroblast cell line, MRC5. (A) Western blot analysis showing protein expression of p21 (21 kDa), p-p21 (Thr145) (32 kDa), and loading control GAPDH (37 kDa) in HL-60 and MRC5. No detectable bands for p21 and p-p21 was observed in the HL-60 cell line. MRC5 was used as a positive control for p21 and p-p21 expression. (B) Representative immunofluorescence images of p21 and p-p21 (Thr145) staining. The HL-60 cell line did not have any detectable p21 or p-p21 staining. Scale bar = 10 µm.
