# Supplementary material for: The cyclin dependent kinase inhibitor p21Cip1/Waf1 is a therapeutic target in high-risk neuroblastoma
Source: Front Oncol. 2022 Sep 6;12:906194. doi: 10.3389/fonc.2022.906194 (PMC9486206; doi:10.3389/fonc.2022.906194)
Supplement: Supplementary file 4 [file Image_3.pdf]

Supplementary Material

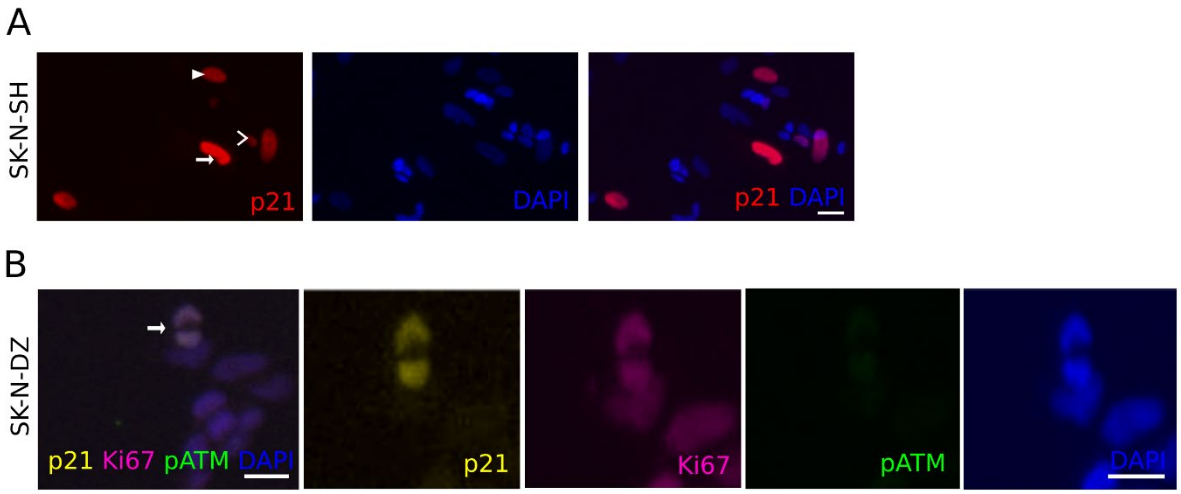

Supplementary Figure 3. Varying endogenous p21 expression and its compatibility with proliferation. (A) Representative image of low (unfilled arrowhead), middle (arrowhead) and high (arrow) endogenous p21 expression shown in the SK-N-SH cell line. Scale bar = 10 µm. (B) Representative images of p21, Ki67 (proliferating) double positive cell (arrow) shown in the SK-N-DZ cell line. Scale bar = 10 µm.
