# Supplementary material for: The cyclin dependent kinase inhibitor p21Cip1/Waf1 is a therapeutic target in high-risk neuroblastoma
Source: Front Oncol. 2022 Sep 6;12:906194. doi: 10.3389/fonc.2022.906194 (PMC9486206; doi:10.3389/fonc.2022.906194)
Supplement: Supplementary file 5 [file Image_4.pdf]

Supplementary Material

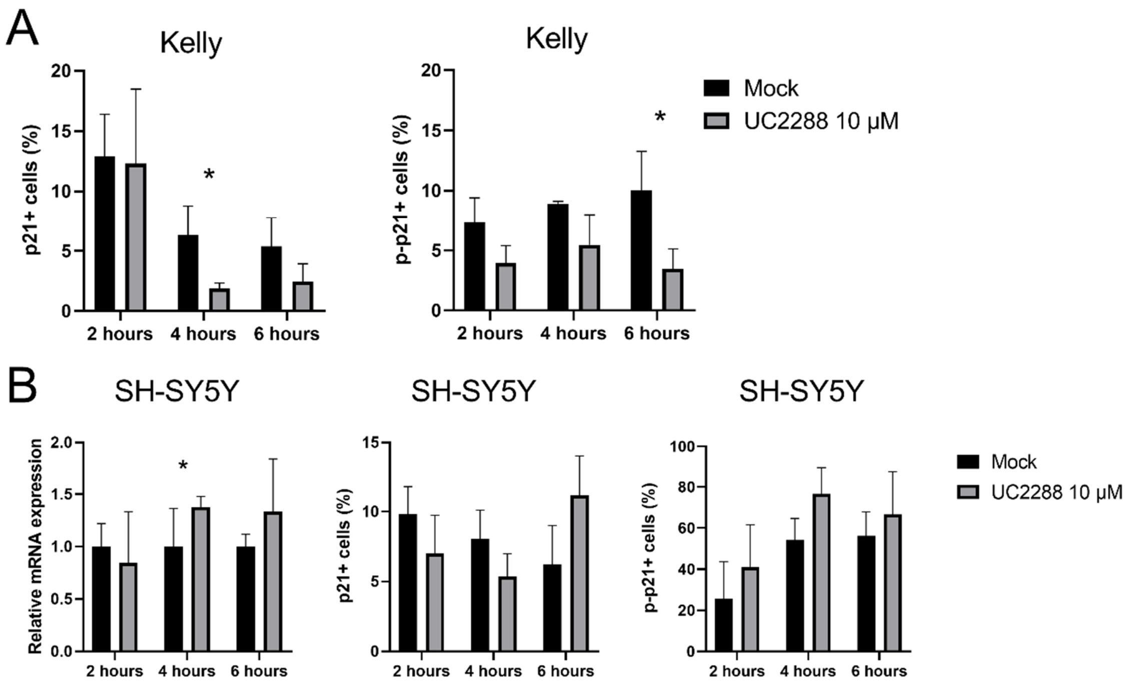

Supplementary Figure 4. p21 inhibition assay using UC2288. (A) p21 inhibition assays performed on Kelly with UC2288 at a concentration of 10 µM at three time points. A decrease in the fraction of p21 positive cells was observed 4 hours after treatment and in the fraction of p-p21 positive cells 6 hours after treatment. * = $p<0.05$, not significant $p>0.05$, Student´s t-test, n=3. (B) p21 inhibition assays performed on SH-SY5Y with UC2288 at a concentration of 10 µM at three time points. An increase in mRNA was observed 4 hours after treatment. No change in either the fraction of p21 or p-p21 positive cells was observed at any of the time points after treatment. * = $p<0.05$, not significant $p>0.05$, Student´s t-test, n=3.
