# Supplementary material for: The cyclin dependent kinase inhibitor p21Cip1/Waf1 is a therapeutic target in high-risk neuroblastoma
Source: Front Oncol. 2022 Sep 6;12:906194. doi: 10.3389/fonc.2022.906194 (PMC9486206; doi:10.3389/fonc.2022.906194)
Supplement: Supplementary file 6 [file Image_5.pdf]

Supplementary Material

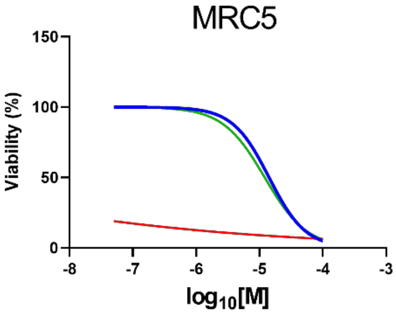

	MRC5
**Cispl**	14.2 µM
**Cispl + UC2288 1 µM**	12.1 µM
**Cispl + UC2288 10 µM**	< 50 nM

Supplementary Figure 5. Viability assay on the fibroblast cell line MRC5. The MRC5 cell line was treatment with cisplatin as a single reagent or in combination with UC2288 (1 µM or 10 µM). A reduction in IC_{50} was observed following combination treatments compared to a single cisplatin treatment. n=3.
