# Supplementary material for: The cyclin dependent kinase inhibitor p21Cip1/Waf1 is a therapeutic target in high-risk neuroblastoma
Source: Front Oncol. 2022 Sep 6;12:906194. doi: 10.3389/fonc.2022.906194 (PMC9486206; doi:10.3389/fonc.2022.906194)
Supplement: Supplementary file 7 [file Image_6.pdf]

Supplementary Material

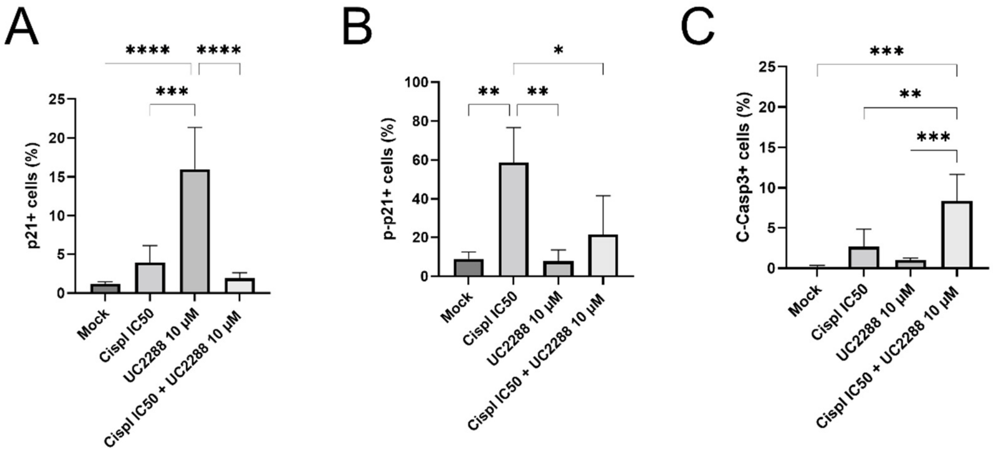

Supplementary Figure 6. Combination treatment with UC2288 and cisplatin in the BE(2)-C cell line. Bar graph showing the fraction of BE(2)-C cells expressing (A) p21, (B) p-p21 (Thr145), and (C) C-Casp3 following 24 hours treatment with Mock, cisplatin at IC_{50}, UC2288 at 10 µM, or a combination of cisplatin IC50 and UC2288 10 µM. Immunofluorescent analysis showed an increase in the fraction of p21 positive cells following treatment with UC2288 at 10 µM, an increase in the fraction of p-p21 positive cells following treatment with cisplatin at IC_{50} and an increase in the fraction of C-Casp3 positive cells following combination treatment. * = p<0.05, ** = p<0.01, *** = p<0.001, **** = p<0.0001, not significant p>0.05. One-way ANOVA with Tukey post hoc test. Mean ± SD, n=4.
